# Differences in use of telemedicine integrated into traditional primary health care – a comparative observational study

**DOI:** 10.1080/02813432.2025.2457542

**Published:** 2025-02-06

**Authors:** Pär Eriksson, Maria Randjelovic, Hans Thulesius, Tora Hammar, Stefan Lagrosen, Evalill Nilsson

**Affiliations:** a Department of Medicine and Optometry, Linnaeus University, Kalmar, Sweden; b Department of Health, Medicine and Caring Sciences, Linköpings Universitet, Linköping, Sweden; c Department of Management, Linnaeus University, Kalmar, Sweden

**Keywords:** Quantitative study, telemedicine, e-consultation, health care seeking behaviour, resource utilisation

## Abstract

Telemedicine in primary health care is expected to address many of the issues currently challenging service delivery. However, the impact and effect will depend on who will use the new technology.

**Objective:**

The objective of the study was to investigate differences between users and non-users of telemedicine integrated into traditional office-based primary health care.

**Methods:**

Quantitative registry-based population study in two regions in the southeast part of Sweden (*n* = 73,486), comparing users with non-users of telemedicine across the variables sex, age, socioeconomic status (SES), morbidity and health care seeking behaviour (HSB). Two study periods of six months were used (September 2019–February 2020 for Region Östergötland, and September 2021–February 2022 for Region Kalmar County) to collect user data. A reference period of 36 months (September 2016–August 2019) was used, to collect data on HSB.

**Results:**

Users were more often women under the age of 60 and had higher morbidity (measured as resource utilisation) than non-users (*p* < .001). In contrast, no statistically significant differences were seen between the two groups regarding SES, measured as Care Need Index (CNI). Regarding HSB, a proxy measure (health record entries) showed more entries for users than non-users.

**Conclusions:**

Our findings suggest that users are more likely to be women and below the age of 60. Likewise, users also tend to have a greater need for health care services compared to non-users, and they seek health care more often compared to non-users. No differences regarding SES were found.

## Introduction

Telemedicine via smartphone or other similar digital devices in primary health care differ from traditional face-to-face consultations in that it offers the possibility for patients and health care personnel to remotely interact. The consultation typically takes place in the form of electronic chat (synchronous or asynchronous) or video meeting, sometimes in combination with automated medical history-taking followed by automated or semi-automated triage [1] International policy institutions’ as well as national governments’ reform agendas reflect expectations about what telemedicine broadly can achieve, such as improving access to care, curbing demand for services, improving quality and boosting organisational efficiency [2–6]. These challenges have been intensified by an ageing population and a need for the provision of continuity of care [7], as well as a need to focus more on the prevention of ill-health and the promotion of healthy lifestyles in the population [8,9]. To a large extent, the development has been driven by private entrepreneurs, with backing from private investors [2,10,11]. In countries such as the UK and Sweden, it has resulted in an disproportionately larger share of consultations with ‘digital-only’ providers, compared to telemedicine integrated into traditional office-based primary health care services, where the majority of the consultations in primary health care takes place [2,11,12].

The impact and effect of the new technology will largely depend on who will use these services. Results reported so far from Sweden indicate that women, younger patients and patients with higher socioeconomic status (SES) are more likely to be users compared to men, older patients and patients with a lower SES, who are more likely to be non-users [13–15]. Additionally, patients with higher health care needs have been found to be less likely to be users of telemedicine services [15], while patients with lower health care needs are more likely to be users [13]. In a study from Canada based on data from 2013 to 2014, McGrail et al. found younger patients (20–44) being more likely to use telemedicine services compared to older patients but found no difference by sex [16]. In a more recent study from the US, Chandrasekaran found significant associations between social determinants, socioeconomic demographics and health factors with telemedicine utilisation. Women were more likely to be users, and socially disadvantaged groups were less likely to be users of telemedicine services [17]. In terms of access to care, some studies suggest that contact using telemedicine technology benefits certain patient groups, for example, patients with mental health issues. For this group, telemedicine offers a choice of contact during the night or over weekends, when the problems can be more intense [18–20]. Benefits have also been found among patients with neuropsychiatric disorders, where anxiety has made them hesitant to contact health care, by phone or in person, also for other health problems [20,21]. On the other hand, telemedicine can also exclude patient groups, for example, the elderly and individuals with limited digital literacy [14,17,22–26].

Contact with a known provider and continuity of care have also been found to be important to the patient when choosing the type of health care contact. Landgren and Cajander [27] found that for elderly people personal contact with a known provider was more important than convenience of care when choosing between face-to-face consultation and telemedicine. McGrail et al. found in a study from Canada that older people and people with numerous health issues had a stronger preference for consulting with a known provider, regardless of form of consultation [16]. Younger individuals did not show this preference.

Traditional primary health care is defined by WHO as the provision of integrated, accessible health care services by practitioners who are accountable for addressing a large majority of personal health care needs [7]. More knowledge is needed about how telemedicine works in traditional primary health care, who the users and non-users are, how the new technology can contribute to addressing identified challenges and be of benefit for both patients and health care staff.

## Aim

The aim of the study was to compare users and non-users of telemedicine integrated into traditional office-based primary health care with regards to demographics, SES, morbidity and health care seeking behaviour (HSB), to better understand the differences and similarities between the two groups.

## Methods

The study design is a quantitative registry-based population study comparing users with non-users of telemedicine across the variables sex, age, SES, morbidity and HSB. SES is measured using the proxy Care Need Index (CNI). HSB is measured using the proxy electronic health record (EHR) entries. HSB in this study is defined as an individual’s propensity to contact primary health care regarding health-related problems.

### Setting

The Swedish health care system is decentralised, organised by 21 independent regional health authorities (regions). Primary health care is provided by multi-professional Primary Health Care Centres (PHCCs) offering a range of services including consultations, diagnostics, treatment and referral to specialist care. The PHCCs are either public (run by the region), or private (contracted by the region). Reimbursement is in both instances based on a combination of capitation and fee-for-service. Based on their preferences, convenience, location or other factors, every resident in Sweden has the right to choose and register with any PHCC within their region. A co-payment is normally charged for each visit for patients above the age of 19 years, currently varying between €10 and €30 depending on region [28]. A high-cost threshold is also in place, currently amounting to €130. This is the highest fee that may be charged over a 12-month period. Due to changes in reimbursement system in early 2016, it became commercially interesting for private providers to offer telemedicine on a large scale to the whole population [2]. It meant that patients could access care from ‘direct-to-consumer’ (DTC) providers and by-pass their PHCC where they were registered to access care. DTC providers are being reimbursed on a fee-per-visit basis via the region where the patient resides. Patient co-payments in the DTC services have varied, but at the start this was a service free of charge for patients, which contributed to the rapid success.

**Figure 1.**
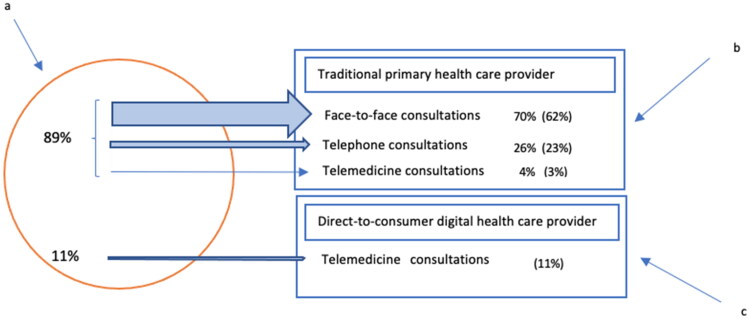
Share of patient–provider consultations in primary health care services in Sweden (traditional primary health care provision and direct-to-consumer (DTC) provision), based on data from 2021/2022, (a), share of traditional primary health care provision (b), share of DTC provision (c), () percent of total volume. Adapted from International Health Policy Survey (IHP) [31] and SALAR [30].

About 90% of the adult population in Sweden is registered with a PHCC [29]. At the time of the study, 89% of the total volume of consultations made by those registered with a PHCC were with PHCCs in the traditional primary health care service, the remaining share were consultations with DTC providers (Figure 1(a)) [29]. Of the consultations made in traditional primary health care office-based face-to-face consultations accounted for roughly 70%, telephone consultations 26% and telemedicine (in this case video consultations) about 4% (Figure 1(b)) [29]. Meanwhile, consultations with DTC providers comprised approximately 11% of total volume of consultations (Figure 1(c)) [30].

The study used data from eight out of 84 PHCCs in two regions in the south-eastern part of Sweden: Region Kalmar County and Region Östergötland. These eight PHCCs were the ones that volunteered to be test pilots for a telemedicine service platform in a joint pilot project within the two regions. Approximately, 80,000 individuals were registered with these PHCCs at the start of the study, equal to just over 10% of the total population in the two regions. The PHCCs represented a mix of public and private providers that offered a combination of telemedicine consultations and office-based visits to its registered population, and operated under similar conditions regarding accessibility, patient fees and provider reimbursement (Table 1).

**Table 1.** Primary Health Care Centre (PHCC) and region.

Primary Health Care Centre and region	Number of registered patients (*n*)	Care Need Index (CNI)	Start of telemedicine pilot project
PHCC1 (Region Östergötland)	12,335	0.87	April 2019
PHCC2 (Region Östergötland)	8,275	0.78	April 2019
PHCC3 (Region Östergötland)	14,471	0.97	April 2019
PHCC4 (Region Östergötland)	14,785	1.29	April 2019
PHCC5 (Region Östergötland)	7,211	0.9	April 2019
PHCC6 (Region Kalmar County)	7,708	0.94	September 2019
PHCC7 (Region Kalmar County)	3,787	1.00	September 2019
PHCC8 (Region Kalmar County)	10,399	1.00	September 2019
Total	78,971		

CNI: Care Need Index.

Number of registered patients at study start. Time of start of telemedicine pilot project.

### Study design

Patient data were collected from eight PHCCs in the two regions Region Östergötland and Region Kalmar County. The two regions border each other geographically and collaborate in the South-Eastern Health care Region collaboration [32]. During 2019, the two regions launched a pilot project offering patients an opportunity to contact primary health care via a telemedicine platform [33]. The platform offered automated triage, asynchronous chat communication, patient upload of photos, and video consultation. Patient-reported measures (questionnaires) could also be answered through the platform.

The pilot was launched in five PHCCs in Region Östergötland starting April 2019, and in three PHCCs in Region Kalmar County starting in September 2019. The two regions collaborated closely during the preparation and launch, which contributed to comparability across the two regions. Initially, a common study period of six months was intended, but due to differences in data registration between the two regions, regarding whether a contact or visit was made through the telemedicine platform or not, this was not possible. By the time, the issue of the data registration was resolved in Region Kalmar County the pilot project in Region Östergötland had already come to an end. Two study periods were therefore used: September 2019–February 2020 (Region Östergötland) and September 2021–February 2022 (Region Kalmar County). Coincidentally, the launch of the pilot also coincided with the outbreak of the COVID-19 pandemic; the first study period took place before the onset of the pandemic and the subsequent restrictions in the population, while the second study period took place after the main COVID-19 vaccination campaigns. Both factors, outside of our control, are very likely to have influenced our results. However, even though there was a difference in time we decided to merge the two datasets. It gave us a broader basis for our study and increased the reliability of the results. The effect of the COVID-19 pandemic was possibly dampened by the fact that the first time-period was before the onset, and the second time-period was after the vaccine campaign; i.e. none of the study periods were during the critical parts of the pandemic. A reference period of 36 months was set to September 2016 through August 2019. The intention of the reference period was to capture HSB among the two study populations. Since the time gap of 18 months between collection of EHR entries theoretically could contribute to a bias in the data material, a *t*-test comparing means between the two regions were performed, which showed no statistically significant difference regarding EHR entries per person (*p* = .07).

**Figure 2.**
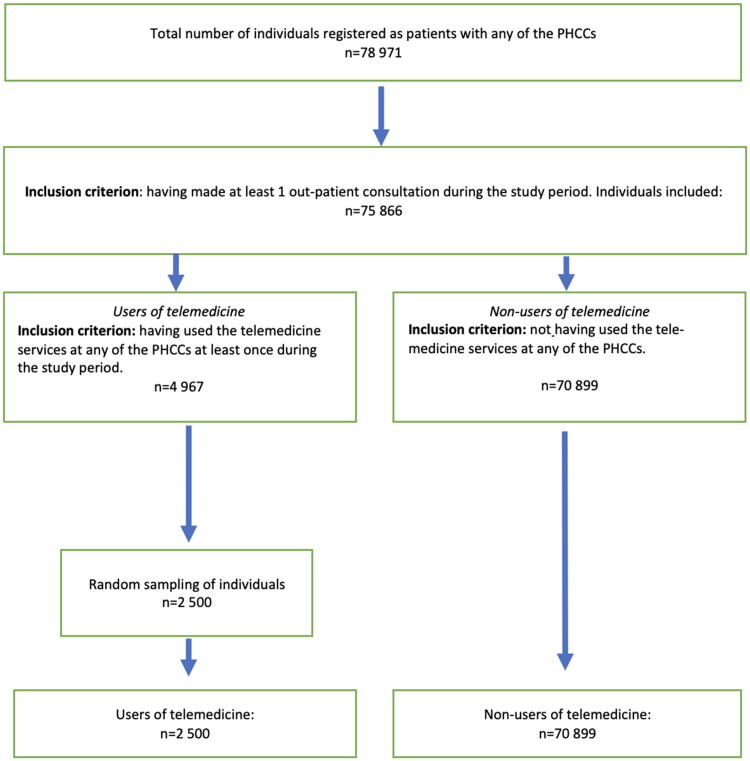
Inclusion criteria of the two populations: users and non-users of telemedicine.

Out of a total number of 78,971 patients registered with the eight PHCCs, two populations were formed. The ‘user’ of telemedicine consisted of a sample of 2500 patients (as advised by the Swedish Ethical Review Authority). These patients were randomly selected from the patients registered at any of the PHCCs at the beginning of the respective study periods. Inclusion criteria were having used the telemedicine service at least once during the respective study periods, in total 4967 patients. Since we in this study were interested in the adoption of a new digital platform, other forms of established contacts, i.e. telephone, e-mail, voice message, which to some extent also can be considered ‘digital’, were not included. The ‘non-users’ of telemedicine consisted of all patients registered with any of the PHCCs at the beginning of the respective study periods. Inclusion criteria were having made at least one PHCC contact during the respective study period but no contact using the telemedicine platform, in total 70,986 patients. To compensate for the limitation in terms of number put on the ‘user’ population, a weight of 1.98 was applied during analysis to each individual included in that group [34]. The population weight was used in the regression analysis. The male to female ratio in the general population was 1.02 [35] (Figure 2).

Assigned clinical groups (ACGs) and Resource Utilisation Band (RUB) formed the basis for assessing morbidity [36]. ACG was derived from EHR data categorising individuals into mutually exclusive clinical groups based on all the diagnoses registered for them during the last 18 months. RUB is a component of the ACG system that further categorises the ACG groups into only six groups (0–5), reflecting their statistically estimated level of resource utilisation regardless of disease or cause of disease. This means that the initial categorisation of individuals according to their current unique health needs (ACG) is followed by a further categorising based on the expected resources these individuals might require (RUB). RUB is used as a basis for resource allocation in primary care in Sweden. Mean value of RUB in the population included in the study was 1.95; standard deviation was 1.3.

The number of EHR entries for each patient registered at any of the PHCCs included in the study at the start of the respective study period was used as a proxy for HSB. These data were retrieved from the local EHR databases. An EHR entry could be notes about the actual meeting between the patient and the health care professionals, but also about referrals, consultations with colleagues, prescriptions, etc. For the present study, it was not possible to retrieve more specific data than this, but even if it might not be the ideal proxy, this inclusion of a broad set of actions reflecting patient–provider interaction was deemed appropriate. A total number of 2,466,911 EHR entries were retrieved over a period of 42 months; the study periods of six months for the respective region, and the reference period (September 2016–August 2019). A three-year period was chosen to allow for a comprehensive view of the patients’ health history and to control for short-term fluctuations in health care utilisation.

The CNI uses socio-economic indicators to identify risk of ill-health among patients registered with a PHCC and constitutes a basis for calculating reimbursements to providers in primary health care in Sweden [37]. In this study, it was used as a proxy for SES. CNI below 0.75 means a low expected care need and corresponds with a high socio-economic status, CNI above 1.25 indicates a high expected care need and corresponds with a low socio-economic status. The socio-economic variables included in the CNI are displayed in Box 1.

Box 1. Socio-economic variables included in the Care Need Index.

**Table:** 

Age over 65 and living alone Born in Africa, Asia, South America or certain countries in southern and eastern Europe Age 16–64 being unemployed or employed in labour market policy measures Single parent with children 17 years old or younger Individual who within the last year moved into the area Individual 26–64 years old with low level of education Age below 5 years old

Data on CNI for each PHCC, computed by sex and age-groups, were obtained by Statistics Sweden. CNI is not available at individual level.

The two populations were divided into five age groups (0–19, 20–39, 40–59, 60–79, 80+), and further stratified by sex. Approval from the Swedish Ethical Review Authority was granted (2020-00486/2021-01253). Structure Query Language (SQL) code used for retrieving data from the EHRs in the two regions is available in Appendix 1 (Table 2).

**Table 2.** Variables and distribution of users and non-users.

	Users *n* = 2500	%	Non users *n* = 70,899	%	Total *n* = 73,399	%
Sex						
Men	993	40%	36,541	51%	37,534	51%
Women	1507	60%	34,445	49%	35,952	49%
Age groups						
0–19	479	19%	14,984	21%	15,463	21%
20–39	978	39%	16,760	24%	17,738	24%
40–59	645	26%	16,846	24%	17,491	24%
60–79	346	14%	17,359	24%	17,705	24%
80+	52	2%	5037	7%	5089	7%
Resource Utilisation Band (RUB)						
0	462	18%	15,803	22%	16,265	22%
1	237	9%	8098	11%	8335	11%
2	617	25%	16,902	24%	17,519	24%
3	995	40%	25,030	35%	26,025	35%
4	139	6%	4075	6%	4214	6%
5	28	1%	991	1%	1019	1%
Missing data	22	1%	87	<1%	109	<1%
Care Need Index (CNI) by sex and age group						
Women 0–19	0.812		0.693			
Women 20–39	1.289		1.227			
Women 40–59	1.063		0.970			
Women 60–79	0.936		1.029			
Women 80+	2.074		1.925			
Men 0–19	0.859		0.700			
Men 20–39	1.135		1.192			
Men 40–59	0.807		0.938			
Men 60–79	0.836		0.273			
Men 80+	1.651		1.074			
Out-patient contacts						
Total number of EHR entries	1,13,305		23,53,606		24,66,911	
EHR entries per patient	45		33		34	

Missing data mean RUB-data for these individuals were not available.

### Statistical analysis

Three models were developed. The first model (Equation (1)) included sex, age and morbidity (RUB) as independent variables to predict the outcome of being a user of telemedicine. The outcome is the binary variable of having used the telemedicine services at least once within the study’s timeframe, or not, classifying individuals as users or non-users. Logistic regression produced odds ratios (ORs) for the independent variables of sex, age and morbidity, adjusting for their potential confounding effects. The first category in each variable was chosen as the reference category, i.e. ‘men’ for sex, ‘0–19’ for age group and ‘RUB = 0’ for morbidity. Subsequent post-estimation analysis was used to calculate the average predicted probability of being a user of telemedicine.

(1) $$\begin{array}{l} \left(\text{User}\right)∼\text{Bernoulli}\left(\theta \right)\text{with log }\text{it}\left(\theta \right)\\ \;=\beta_{0}+\beta_{1}\times_{.}\left(\text{Sex}\right)+\beta_{2}\times (\text{Age})+\beta_{3}\times \left(\text{Morbidity}\right) \end{array}$$

An OR greater than 1 indicates a positive association between the predictor and the outcome, while an OR below 1 indicates a negative association between the predictor and the outcome.

Equation (2) uses an independent *t*-test to compare averages of CNI between the two groups of users and non-users of telemedicine. Since CNI data were only obtained at population level (stratified by age group and sex), it was excluded from the regression model (Equation (1)). Instead, to assess differences, mean values along with their confidence intervals were computed for both populations.

(2) $$\left(\text{CNI}\right)∼\text{Normal}(\mu ,\sigma )\text{ with }\mu =\beta_{0}+\beta_{1}\times \left(\text{User}\right)$$

In the study, the number of EHR entries was assumed to follow a Poisson distribution and modelled using Poisson’s regression. Equation (3) was formulated to estimate the incidence rate ratio (IRR) assessing the likelihood of individuals being classified as users or non-users of telemedicine. This analysis facilitated a comparison between the two populations with respect to the frequency of EHR entries, used as a proxy for HSB.

(3) $$\left(\text{EHR entries}\right)∼\text{Poisson}\left(\lambda \right)\text{ with log}\left(\lambda \right)=\beta_{0}+\beta_{1}\times \left(\text{User}\right)$$

To further clarify the distribution of RUB values in the populations, an independent *t*-test was used to compute means.

All analyses were conducted in STATA 18 (StataCorp, College Station, TX).

## Results

The results (Supplementary Table 4) suggest that women were more likely to be users of telemedicine compared to men (*p* < .001). Furthermore, the results suggest that patients belonging to age group 20–39 were more likely to be users of telemedicine services compared to the reference group (*p* < .001). Age groups 60–79 and 80+ were less likely to be users of telemedicine compared to the reference group (*p* < .001). Patients belonging to RUB groups 2–5 were more likely to be users of telemedicine compared to the reference (*p* < .001). The mean RUB value in the two populations was computed to 2.1 among users and 1.9 among non-users (*p* < .001). Furthermore, the results suggest a positive relationship between the number of EHR entries and the likelihood of being a user of telemedicine, with an IRR of 1.5 (*p* < .001). Regarding CNI, the *t*-test did not indicate any statistically significant differences between users and non-users of telemedicine (Table 3).

**Table 3.** Odds ratio estimates of the odds, and incidence rate ratio, of having made at least one consultation using the telemedicine platform among registered patients at eight Primary Health Care Centres (PHCCs) in Region Kalmar County and Region Östergötland during the study periods.

Variable	Measure	95% confidence interval	*p* Value
Sex	Odds ratio (OR)	CI	*p*
Men (reference)			
Women	1.39	1.28–1.51	<.001
Age-groups, years	OR	CI	*p*
0–19 (reference)			
20–39	1.64	1.51–1.78	<.001
40–59	1.00	0.92–1.11	.850
60–79	0.45	0.40–0.50	<.001
80+	0.19	0.16–0.25	<.001
Age-groups, years	Average predicted probability	CI	*p*
0–19	0.07	0.06–0.07	<.001
20–39	0.11	0.10–0.11	<.001
40–59	0.07	0.06–0.07	<.001
60–79	0.03	0.03–0.04	<.001
80+	0.01	0.01–0.02	<.001
Resource Utilisation Band (RUB)	OR	CI	*p*
RUB 0 (reference)			
RUB 1 vs. RUB 0	0.93	0.82–1.04	.213
RUB 2 vs. RUB 0	1.23	1.13–1.35	<.001
RUB 3 vs. RUB 0	1.76	1.62–1.91	<.001
RUB 4 vs. RUB 0	2.09	1.81–2.42	<.001
RUB 5 vs. RUB 0	2.67	2.02–3.54	<.001
RUB *t*-test	Mean	CI	*p*
User	2.07	2.03–2.12	
Non-user	1.94	1.94–1.96	<.001
Resource Utilisation Band (RUB)	Average predicted probability	CI	*p*
RUB 0	0.05	0.04–0.05	<.001
RUB 1	0.04	0.04–0.05	<.001
RUB 2	0.06	0.05–0.06	<.001
RUB 3	0.08	0.08–0.09	<.001
RUB 4	0.09	0.09–0.10	<.001
RUB 5	0.12	0.09–0.15	<.001
CNI *t*-test	Mean	CI	*p*
Users	1.14	0.82–1.32	
Non-users	1.07	0.84–1.45	.671
EHR entries per patient	Incidence rate ratio (IRR)	CI	*p*
Non-users (reference)			
Users	1.5	1.32–1.72	<.001

Mean values of RUB and CNI comparing users with non-users. Post-estimation of average predicted probability by age group and RUB. Incidence rate ratio of EHR by user and non-user.

**Figure 3.**
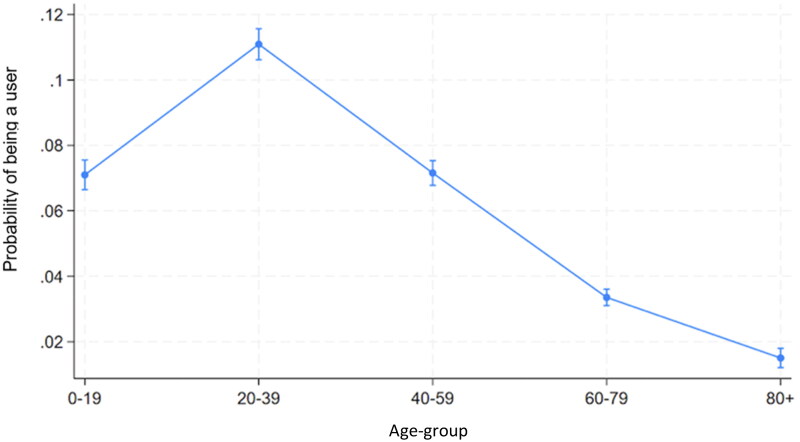
Predictive margins of being a user of telemedicine services by age-groups with 95% confidence intervals

Post-estimation indicates the predicted probabilities of the outcome variable. The lowest predicted probability of being a user was noted among age-groups 60–79 and 80+. The highest predicted probability is noted in age-group 20–39. The differences are statistically significant (Figure 3).

**Figure 4.**
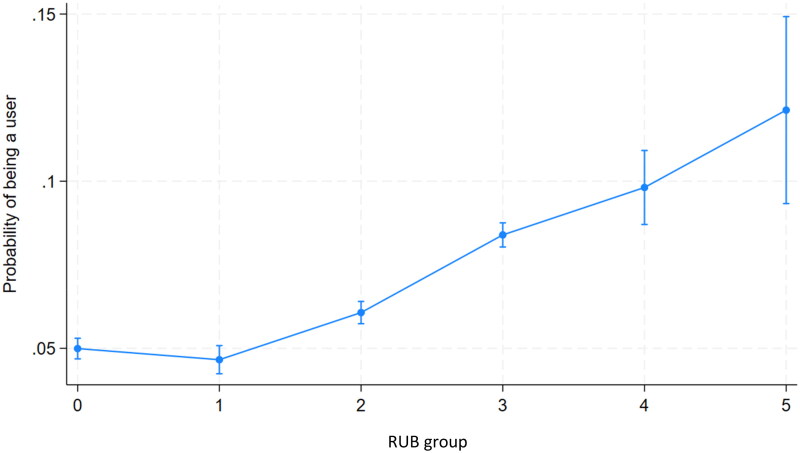
Predictive margins of being a user of telemedicine services by RUB group with 95% confidence intervals.

A similar analysis of RUB groups indicated a higher predicted probability among patients belonging to RUB groups 2, 3, 4 and 5 to be users. However, the confidence intervals for RUB groups 4 and 5 are wide, and therefore the difference among these groups is not statistically significant (Figure 4).

## Discussion

The aim of the study was to investigate differences between users and non-users of telemedicine integrated into traditional office-based primary health care. Our findings show that users are more likely to be women and below 60 years of age. Additionally, users tend to have a higher morbidity and may also be seeking health care more often. However, when it comes to differences regarding SES, we did not find any statistically significant differences in CNI between the two groups.

Our results suggest that a scale-up of telemedicine in traditional primary health care has the potential to attract a broad set of patients, especially when compared to patient profiles among DTC providers [13,14]. Our findings suggest that users are predominately the young (0–19 years and 20–39 years) and the middle-aged (40–59 years) patients. In addition, the probability of being a user seems to be slightly higher among patients with higher morbidity (RUB 3–5) compared to patients with lower morbidity (0–2), as displayed in Figure 4. These are interesting findings since other studies, not least regarding DTC providers, report users of telemedicine mostly being associated with younger age groups (<40 years), and predominantly ‘easy cases’ linked to relatively low morbidity rates [13,30,38]. Even though the EHR entries, as described above, comprise many different things it could be argued that the higher number of EHR entries among users would indicate that this group seeks health care more often than non-users. For frequent users of health care, the option of using telemedicine consultation services with a known provider (traditional primary health care) could potentially mean an improvement in terms of better access and a more efficient handling of the case, which potentially could reduce the overall number of contacts needed compared to if telemedicine was not available [18]. This is perhaps what has been reported in some qualitative studies regarding patients suffering from anxiety and other mental health problems, or patients suffering from chronic conditions, and their access to care through telemedicine [18,21,39], but also among marginalised groups having previous negative experiences from health care encounters, or stigma [19]. However, the degree to which telemedicine substitutes for other visits, as opposed to generating additional demand and escalating the workload at the PHCC, remains to be further investigated.

Our data show that patients with higher RUB-values seem to be more likely to be users of telemedicine integrated into traditional office-based primary health care compared to patients with lower RUB values (Figure 4), suggesting that users of telemedicine possibly also are patients with higher morbidity. The RUB *t*-test also displays a small but statistically significant difference in means, which is higher for users compared to non-users. In part, but not entirely, that difference could be explained by a higher number of EHR entries for the group of users since ACG is based on registered diagnoses. Those who are ill but do not contact the health care system will have a lower ACG/RUB, since there will be no trace of the illness in their EHR. On the other hand, the higher number of EHR entries could also in part be explained by greater morbidity, since very ill people would be more likely to seek health care, rendering an entry in their EHR, typically patients with a chronic disease. It is not possible to detect to what extent these correlations influence our findings, since the variables are interdependent. However, the great difference between users and non-users in the number of EHR entries per patient is not likely to be entirely explained by the difference in morbidity between the two groups and would rather suggest that users have a greater tendency to seek health care assistance. In either case, our findings differ from those of earlier studies that suggest that determinants of the use of telemedicine are generally not associated with greater health care needs [13,15].

In line with our findings, Reed et al. [26] report that young and middle-aged patients are more inclined to use telemedicine compared to older patients. Reasons for non-use of telemedicine services among the elderly have been reported to be linked to personal interaction and continuity of care. For example, having access to a known provider seems to be more important for this patient group than other potential conveniences when choosing the type of contact, for example via telemedicine platform [27]. Therefore, scaling up telemedicine in traditional primary health care needs to consider preferences among older people currently being reluctant or not able to use these services. More evidence about how to best provide telemedicine in combination with continuity of care for these patient groups, and how it can contribute to efficiency gains in service delivery, is of need.

In our study, we were not able to establish any association between SES and the use or non-use of telemedicine. These findings are contradictory to what has been reported in several other studies where users of telemedicine have been associated with a higher SES, compared to non-users [14–16,40]. One possible interpretation is that telemedicine, when integrated into the traditional primary health care, attracts a wider group of users, which therefore contributes to diminishing socio-economy disparities between users and non-users. This is an important finding, since it reduces the likelihood of telemedicine disproportionately favouring those with a stronger socio-economic background at the expense of socio-economically weaker groups. Our results suggest that such an imbalance could be eliminated, or at least be less pronounced, when the services are provided in a traditional office-based primary health care setting. Nevertheless, there might still be other socio-economic disparities that could contribute to differences in the use of telemedicine which we have not been able to capture in our study, and these must be identified and addressed when scaling up these services in primary health care.

## Strengths and limitations

The main strength of this study is the traditional office-based primary health care setting as opposed to the DTC provision of care which has so far mainly been studied in Sweden. This focus provides deeper insight into how telemedicine functions in settings where in fact most consultations in primary health care occur. The quantitative study design complements several earlier, qualitative studies of telemedicine in traditional primary health care.

Measuring differences in use of telemedicine is to a large extent confined by the data available from patients contacting a health care provider regarding a health problem. Information from the population in general is lacking, at least in our study, and therefore the validity could be questioned as only measuring different aspects of morbidity. Another aspect that we are not capturing with our model is the effect of early adopters which could lead to both an overestimation and an underestimation of results. The results therefore need to be interpreted with some caution. Combining qualitative and quantitative approach can add additional insights and improve the validity.

A major limitation of the study is that we were not able to fit a complete model for the analysis, instead we had to fit separate models, one model for sex, age and RUB, a separate model for CNI, and a separate model for HSB. The data material we had access to did not allow the use of a single model in which all variables were included. The results should be interpreted with this in mind.

It is difficult to determine if and how the use of different time periods for the two study populations has affected the results of the study. The study period happened to coincide with the covid-19 pandemic, when telemedicine was a necessity rather than a choice, introducing more people to digital health care and possibly inducing new behaviours regarding how patients choose to contact care even after the pandemic. However, we found no differences in EHR records between the two study populations. Additionally, local health care statistics show that the increase in telemedicine during the pandemic was temporary, as post-pandemic use of telemedicine is similar to pre-pandemic levels, disappointing regional leaders who had hoped the pandemic would change the way patients permanently seek care towards more telemedicine [41]. Furthermore, the combination of data from PHCCs in two different regions using the same digital platform improves both generalisability and consistency of the results. The larger sample from a larger group of PHCCs has therefore contributed to the robustness of our results.

The fact that the sample of PHCCs is restricted to units that were piloting the telemedicine platform could potentially cause a bias as the PHCCs all volunteered to participate in the pilot, and therefore potentially had a more positive and facilitating attitude towards the new technology being introduced. Another limitation is the short time period of six months, which can also have affected the outcome. It should also be noted that the data that were available for this study were dependent on the content of the EHR databases in the respective region. Even if the same EHR system is used in both regions, data entry procedures may differ, placing limits on how data can be pooled for analysis. Finally, there are alternatives to measure SES other than CNI that could have been used. However, they would have had to be retrieved from other data repositories not accessible for this study.

## Conclusions

The aim of the study was to investigate differences between users and non-users of telemedicine integrated into traditional primary health care. Our findings suggest that users are more likely to be women and below the age of 60. Users also tend to have a greater need for health care services compared to non-users, and usage of integrated telemedicine does not seem to be associated with lower morbidity. However, when it comes to differences regarding SES, we did not find any statistically significant differences between the two groups.

## Supplementary Material

Supplemental Material

## Data Availability

Data are available upon request.
