# Supplementary material for: Differences in use of telemedicine integrated into traditional primary health care – a comparative observational study
Source: Scand J Prim Health Care. 2025 Feb 6;43(2):476–87. doi: 10.1080/02813432.2025.2457542 (PMC12090274; doi:10.1080/02813432.2025.2457542)

**Supplements**

Table 4: *Odds ratio estimates of the odds, and incidence rate ratio, of having made at least one digital consultation among registered patients at eight primary health care centres (PHCC) in Region Östergötland during the study periods.*

**
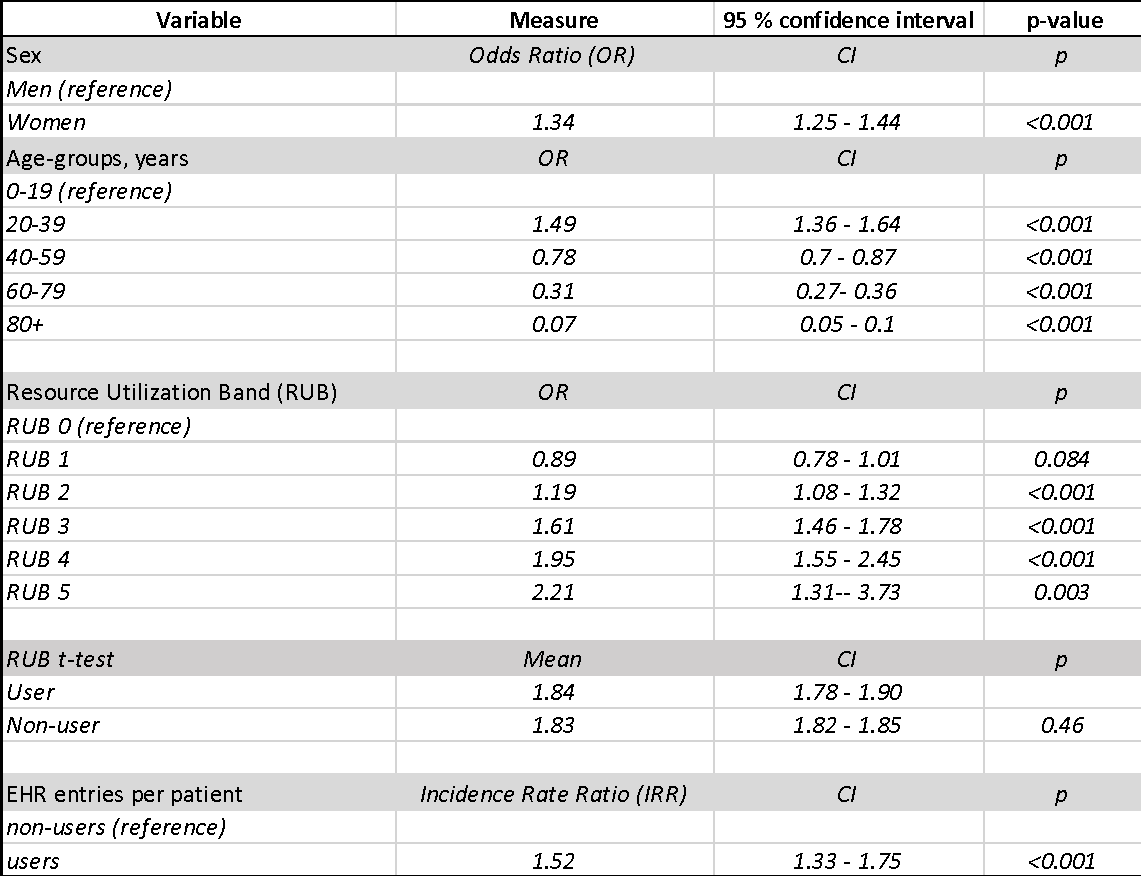
**

Table 5: *Odds ratio estimates of the odds, and incidence rate ratio, of having made at least one digital consultation among registered patients at eight primary health care centers (PHCC) in Region Kalmar län during the study periods.*


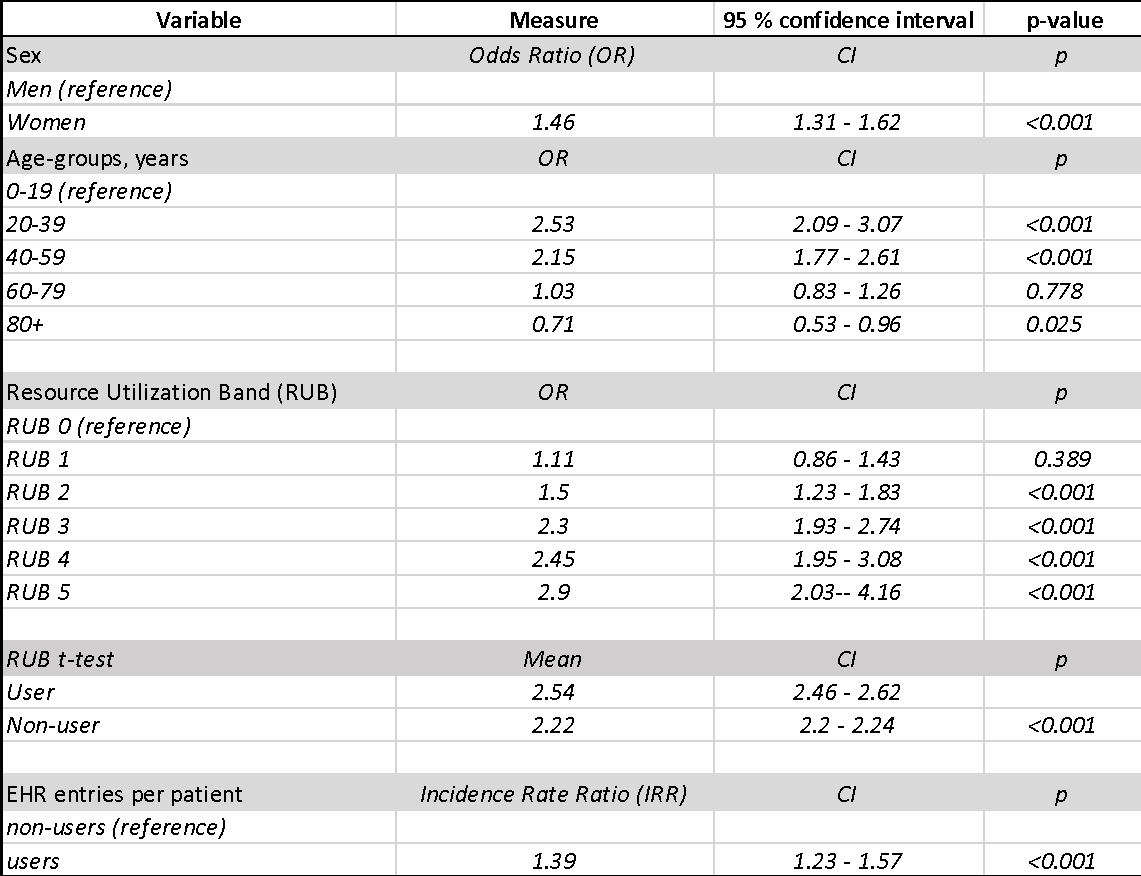


Table 6: Study population and reference population by county, sex, age-group, RUB and EHR entries


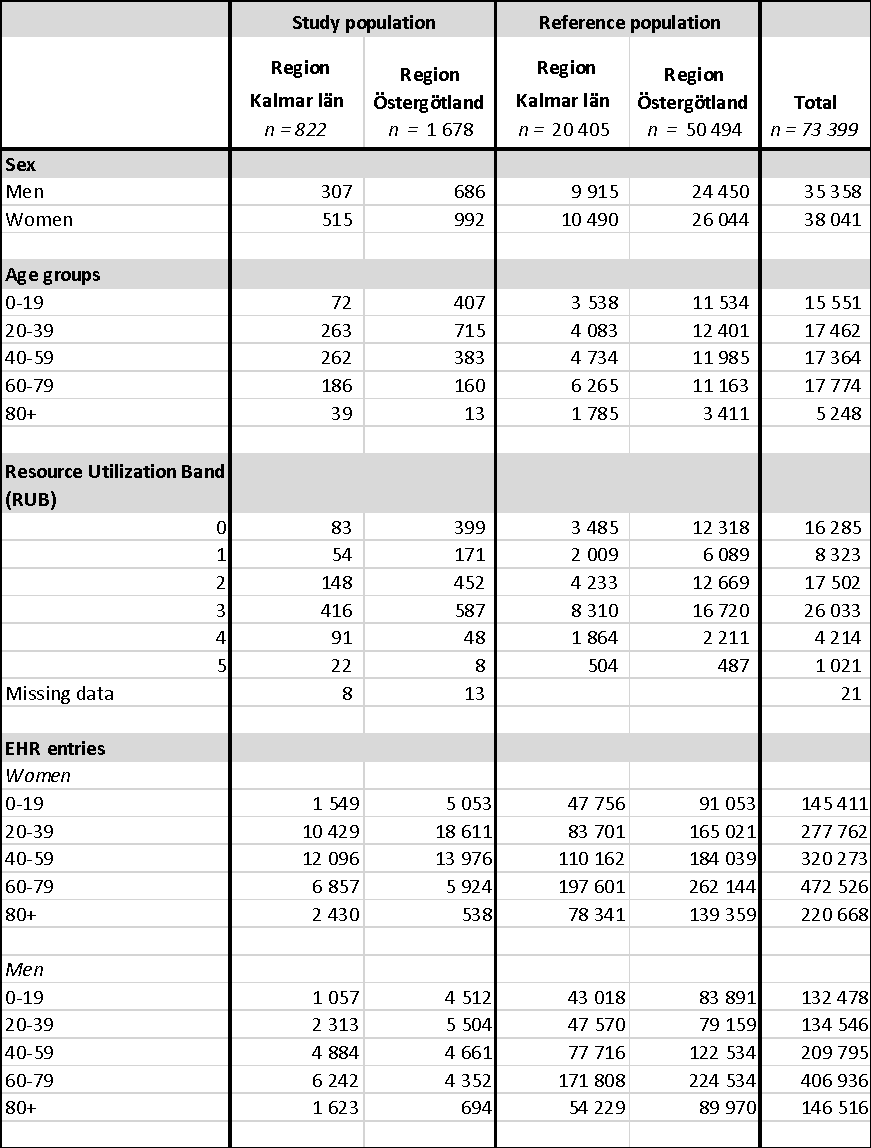

Supplement: Supplemental Material [file IPRI_A_2457542_SM6991.docx]
